# 116 Does Insurance Status Affect Outcomes in Burn Admissions?

**DOI:** 10.1093/jbcr/iraf019.116

**Published:** 2025-04-01

**Authors:** Arman Fijany, Tyler Murphy, Emily Swafford, Punit Vyas, Jordan Garcia, Robel Beyene, Stephen Gondek, Anne Wagner, Elizabeth Slater

**Affiliations:** Vanderbilt University Medical Center; Vanderbilt University Burn Center; Vanderbilt University Burn Center; Vanderbilt University Medical Center; Vanderbilt University Medical Center; Vanderbilt University Medical Center; Vanderbilt University Medical Center; Vanderbilt University Medical Center; Vanderbilt University Medical Center

## Abstract

**Introduction:**

Burn injuries can require extended hospital stays, costly procedures, and intensive physical and occupational therapy after discharge. Lack of insurance or access to healthcare can frequently influence outcomes for these patients. Our objective was to compare outcomes for patients admitted to burn centers with and without insurance.

**Methods:**

The American Burn Association (ABA) Noncommercial Burn Research Dataset was queried to search for admissions that included insurance status over ten years beginning in 2012. Demographic data and discharge disposition were recorded and analyzed with the chi-square test. Mortality was the primary outcome with ICU admission, respiratory failure, deep venous thrombosis (DVT), and hospital length of stay (LOS) were analyzed as secondary outcomes. Renal failure, unplanned intubation, cellulitis, systemic sepsis, and alcohol withdrawal were tertiary outcomes. Primary and secondary outcomes underwent covariate logistic regression analysis – adjusted for age, sex, TBSA%, and inhalation injury – to reduce the effects of these confounders. The Python language and PyCharm 3.1 software with Pandas, NumPy, and SciPy. Stats modules were used for all data analysis.

**Results:**

34,441 burn admissions were classified as uninsured compared to 74,886 with private insurance. Those uninsured had roughly three times higher odds of leaving against medical advice (AMA, OR = 2.97, p < 0.0001). Those who did not have insurance were significantly less likely to be white, more likely to be male, older (35.35±16.44 vs. 33.76±21.52), and have a flame injury (p < 0.0001 for all). Lack of insurance was associated with fewer unplanned intubations (p < 0.01), but an increase in alcohol withdrawal was seen in those without insurance (p < 0.0001). After removing the effects of covariates, uninsured status was linked to fewer ICU admissions (OR = 0.92), 1.06 days shorter hospital (LOS), higher rates of respiratory failure (OR = 1.61), and higher mortality rates (OR = 1.81, p < 0.0001 for all).

**Conclusions:**

Patients lacking insurance are roughly three times as likely to leave AMA. Lack of insurance is also associated with significantly higher rates of alcohol withdrawal and mortality while also being correlated with significantly lower ICU admissions and shorter hospital stays. This nationwide database assessment is the most extensive analysis of insurance status on burn admissions.

**Applicability of Research to Practice:**

These findings elucidate the disparity in outcomes for those with decreased access to healthcare. Burn centers and clinicians could potentially enhance case management services to those without insurance to reduce the likelihood of leaving AMA or have increased vigilance for these populations to avoid alcohol withdrawal.

**Funding for the Study:**

N/A

